# Comment on David Haig’s ‘Troubled sleep’: Implications for functions of infant sleep

**DOI:** 10.1093/emph/eou009

**Published:** 2014-04-08

**Authors:** Patrick McNamara

**Affiliations:** Department of Neurology, A9-45, Boston University School of Medicine, and VA New England Healthcare System, Graduate School, Northcentral University, USA

**Keywords:** infant sleep, attachment, REM, genomic imprinting

My comments on Haig’s updating of Blurton Jones and da Costa’s [1] hypothesis on the effects of infant night wakings (i.e. that night wakings function to prolong inter-birth intervals through nursing-induced suppression of ovulation) will focus on its implications for infant sleep architecture and sleep function in both infants and adults more generally. I will also argue that taking into account the ‘attachment status’ of the infant–mother dyad will strengthen the hypothesis as attachment orientation strongly modulates night wakings in the infant. Attachment behaviors should not be construed as exempt from parent offspring conflict. Instead, they should be seen as strategic responses by both parties to that conflict. Attention to these details will strengthen the hypothesis in my view.

Haig’s hypothesis predicts that ‘Maximal night-waking … will … overlap with the greatest benefits of contraceptive suckling. Consistent with this expectation, infant sleep becomes more fragmented after six months and then gradually consolidates.’ Night wakings and sleep consolidation, however, are known to follow significantly different courses during the first year of life dependent on attachment classifications of the infants. For example, McNamara *et al.* [2] reported that at 15 months of age infants with insecure-resistant attachments evinced significantly greater numbers of night wakings and longer mean durations of night-waking episodes than their insecure-avoidant counterparts. Beijers *et al.* [3] recently reported similar findings with infants with an insecure-resistant attachment at 12 months of age awaking more during the night in their first 6 months of life as compared with infant with an insecure avoidant orientation who awoke least often among all other infants. If night wakings decline at a slower rate for insecure resistant versus avoidant infants then the point at which maximal benefits of contraceptive suckling are obtained may differ for infants dependent upon attachment orientation.

It may be that infants with insecure avoidant orientations adopt a strategy of NOT attempting to prolong IBI so that the Blurton-Jones/daCosta/Haig hypothesis applies only to infants with insecure resistant orientations. Optimal IBIs may differ for mother–infant dyads depending on both attachment status and ecologic context. In environments with high mortality rates parents should pursue an opportunistic reproductive strategy aimed at greater numbers of offspring appearing at shorter IBIs and receiving lower levels of investment (e.g. reduced nursing) resulting in fewer night wakings and greater numbers of avoidant attachment orientations.

Most night wakings emerge from active sleep o*r REM* [4–10]. If night wakings are influenced, as Haig argues, by genes of paternal origin, then it is reasonable to suggest that AS/REM might also be differentially influenced by genes of paternal origin. McNamara *et al.* [11, 12] made this case independently based on functional associations and properties of REM. In infant monkeys, AS/REM intensity at first increases and then declines or disappears entirely after prolonged separation from the mother [13–15]. Infant rats appear to suckle while in REM and their REM-related suckling pattern induces milk ejections (via oxytocin release) in the mother [16–20]. The suckling pups stay in REM until they have extracted milk equivalent to 4% of their body weight [21, 22]. When recorded polygraphically, AS/REM sleep is characterized by a low voltage, fast electroencephalogram (EEG), widespread brain activation and arousal, particularly in amygdalar and limbic brain sites subserving aggression, rapid and irregular heart and respiratory rates, penile erections in males (even in infants), and rapid saccadic eye movements under closed lids. In addition, suckling responses, facial grimaces, smiles, cries and other vocalizations and distal limb twitches can be observed. In contrast, NREM sleep is characterized by slow, synchronized, high-voltage EEG patterns, regular and slowed respiratory and heart rates, few or no vocalizations and the absence of eye movements and other peripheral body movements.

Assuming that AS/REM is differentially influenced by genes of paternal origin then both REM properties and REM-associated awakenings can be better explained by mechanisms of genomic conflict than by traditional claims that REM functions as an anti-predator ‘sentinel’ for the sleeping organism. Capellini *et al.* [23], using phylogenetic comparative methods on a large dataset of REM and NREM sleep quotas from a variety of mammalian species found that neither REM sleep quotas nor sleep-cycle length (associated with arousals/awakenings) were significantly associated with three different measures of predation risk, undermining the idea that REM and REM awakenings are anti-predator adaptations.

If properties and functions of AS/REM are better explained as due to the influence of genes of paternal origin (rather than overall protection of the organism), then AS/REM sleep in the infant should, according to genomic conflict theory, function to extract resources from the mother consistent with getting paternal line genes into the next generation. As we have seen REM is associated with night wakings and suckling behaviors in the infant. Although REM percent of total sleep declines with age it nevertheless persists into the adult stage. What about the functions of REM sleep in the adult? REM indices appears to vary with attachment (reproductive) strategies in the adult [24] but little else is known concerning putative functions of REM. Many investigators have noted the paradoxical properties of REM and concluded it has no function at all [12] but if some continuity can be expected from developmental AS/REM into adult REM then it is reasonable to hypothesize REM involvement in reproductive strategies and sexual conflict.

**Conflict of interest:** None declared.
